# Tomato Sterol 22-desaturase Gene *CYP710A11*: Its Roles in *Meloidogyne incognita* Infection and Plant Stigmasterol Alteration

**DOI:** 10.3390/ijms232315111

**Published:** 2022-12-01

**Authors:** Alessandro Cabianca, Andrea Caroline Ruthes, Katharina Pawlowski, Paul Dahlin

**Affiliations:** 1 Entomology and Nematology, Plant Protection, Agroscope, Müller-Thurgau-Strasse 29, 8820 Wädenswil, Switzerland; 2 Mycology, Plant Protection, Agroscope, Müller-Thurgau-Strasse 29, 8820 Wädenswil, Switzerland; 3 Department of Ecology, Environment and Plant Sciences, Stockholm University, 106 91 Stockholm, Sweden

**Keywords:** plant parasitic nematode, β-sitosterol, stigmasterol, *CYP710A11*, sterol C-22 desaturase mutant

## Abstract

Sterols are isoprenoid-derived lipids that play essential structural and functional roles in eukaryotic cells. Plants produce a complex mixture of sterols, and changes in plant sterol profiles have been linked to plant–pathogen interactions. β-Sitosterol and stigmasterol, in particular, have been associated with plant defense. As nematodes have lost the ability to synthesize sterols de novo, they require sterols from the host. Tomato (*Solanum lycopersicum*) plants infected by the plant parasitic nematode *Meloidogyne incognita* show a reduced level of stigmasterol and a repression of the gene *CYP710A11*, encoding the sterol C-22 desaturase that is responsible for the conversion of β-sitosterol to stigmasterol. In this study, we investigated the role of the tomato sterol C-22 desaturase gene *CYP710A11* in the response to infection by *M. incognita*. We explored the plant–nematode interaction over time by analyzing the plant sterol composition and *CYP710A11* gene regulation in *S. lycopersicum* after *M. incognita* infection. The temporal gene expression analysis showed that 3 days after inoculation with *M. incognita*, the *CYP710A11* expression was significantly suppressed in the tomato roots, while a significant decrease in the stigmasterol content was observed after 14 days. A *cyp710a11* knockout mutant tomato line lacking stigmasterol was analyzed to better understand the role of CYP710A11 in nematode development. *M. incognita* grown in the mutant line showed reduced egg mass counts, presumably due to the impaired growth of the mutant. However, the nematodes developed as well as they did in the wild-type line. Thus, while the suppression of *CYP710A11* expression during nematode development may be a defense response of the plant against the nematode, the lack of stigmasterol did not seem to affect the nematode. This study contributes to the understanding of the role of stigmasterol in the interaction between *M. incognita* and tomato plants and shows that the sterol C-22 desaturase is not essential for the success of *M. incognita*.

## 1. Introduction

**Figure 1:**
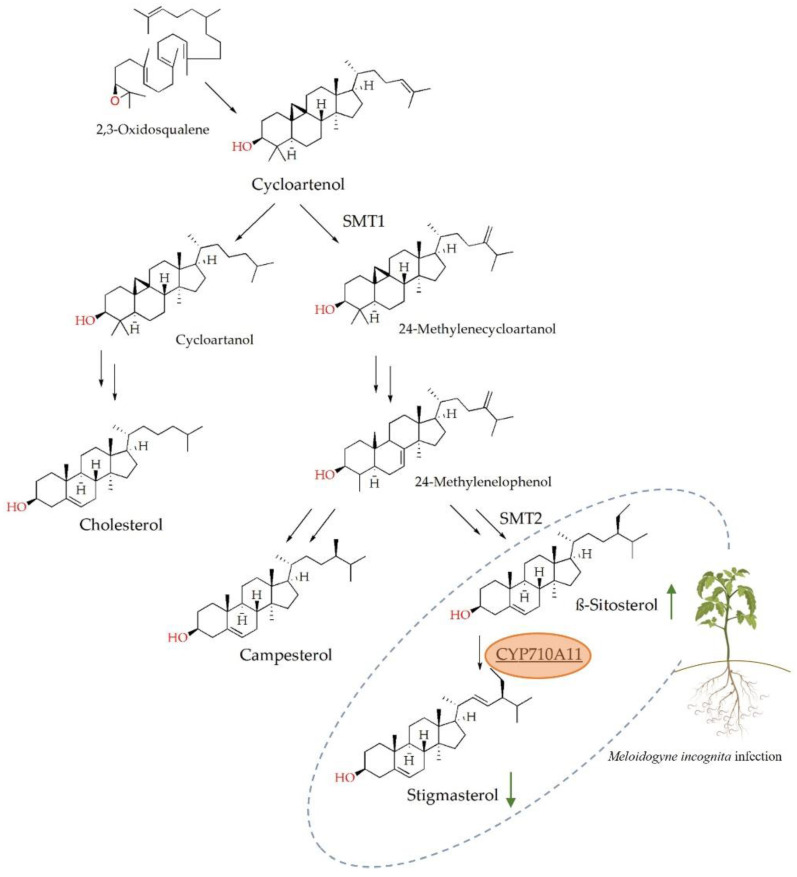
Cycloartenol sterol biosynthesis pathway up to the main plant sterols, campesterol, β-sitosterol, stigmasterol and cholesterol. The biosynthesis pathway of the C-24 alkyl phytosterols is characterized by the alkylation of the side chain by sterol methyltransferase (SMT) 1 and 2. The synthesis of stigmasterol from β-sitosterol is catalyzed by a single enzymatic reaction of a sterol C-22 desaturase in *Solanum lycopersicum* by the cytochrome P450 710A11 (CYP710A11) enzyme (highlighted in orange). Single arrows indicate one enzymatic step, while double arrows indicate multiple enzymatic steps. The green arrows show the changes in the β-sitosterol to stigmasterol ratio caused by *M. incognita* infection and are represented in the gray dotted area.

Sterols are isoprenoid-derived lipids that are essential for multicellular organisms, as they play important roles in regulating membrane fluidity, permeability and function in multiple cellular processes [1,2]. Sterol biosynthesis is a multistep process catalyzed by a series of mostly conserved enzymes (Figure 1) [3,4]. Phylogenetic studies have revealed that sterol biosynthesis evolved early during evolution. Despite the physiological importance of sterols, gene losses have resulted in sterol auxotrophy in multiple lineages, such as nematodes, insects and oomycetes [5,6,7,8].

Due to the importance of these molecules, nematodes must obtain sterols from their diet. A sterol deficiency in *Caenorhabditis elegans*, the model nematode, causes the larvae to remain in the “dauer” stage, failing to transform into the adult stage [7].

Unlike animals and fungi, where cholesterol and ergosterol, respectively, are the most abundant sterols, plants possess a variety of sterols, such as β-sitosterol, campesterol and stigmasterol [9]. Plant sterols differ structurally from cholesterol in the presence of an additional methyl or ethyl group at C-24, which is transferred by the sterol methyltransferases SMT1 and SMT2, and the stigmasterol has an additional double bond at C-22, introduced by the sterol C-22 desaturase (Figure 1) [9,10].

Changes in phytosterol abundance during infection by the plant parasitic nematode *Meloidogyne incognita* have been observed in different hosts, such as *Cucumis sativus* (cucumber), *Glycine max* (soybean), *Solanum lycopersicum* (tomato), *Zea mays* (corn) and *Gossypium hirsutum* (cotton), suggesting the possible role of these molecules in plant defense against nematodes [11,12]. The main effect of *M. incognita* infection on the sterol levels was a change in the β-sitosterol/stigmasterol ratio, β-sitosterol increased and stigmasterol decreased [11,12]. These two molecules are the end sterols of one of the branches of phytosterol biosynthesis, where stigmasterol is converted into β-sitosterol by the sterol C-22 desaturase (CYP710, EC: 1.14.19.41), which introduces a double bond at position C-22 [13] (Figure 1). CYP710 belongs to the cytochrome P450 (CYP) superfamily, representing the largest family of enzymes in plants that are essential for numerous biosynthetic pathways vital for plant development and involved in plant stress responses [14]. It is assumed that CYP710 evolved from the CYP51 family, which comprises the sterol modification enzyme sterol 14-demethylase (EC 1.14.13.70). Arabidopsis and rice plants have multiple copies of the *CYP710A* gene, whereas tomato has a single copy of *CYP710A11* [13].

To date, little is known about the regulation of sterol biosynthesis during nematode infection and the role of *CYP710A11*. However, in tomato, the expression of the *CYP710A11* gene has been shown to be suppressed by the infection of the economically important plant parasitic nematode *M. incognita* [12].

*Meloidogyne* species, also called root knot nematodes, are obligate endoparasites that can cause severe crop damage, even leading to total crop loss [15]. *M. incognita* second-stage juveniles (J2) penetrate the root tip and migrate intercellularly towards the elongation zone, where they induce the formation of a nematode feeding site consisting of giant cells. These giant cells cause root galling and function as the only nutrient supply throughout the nematode life cycle. They are essential for successful nematode reproduction [16,17]. An analysis of cucumber and tomato infected by *M. incognita* revealed that changes in the β-sitosterol/stigmasterol ratio were even more pronounced in the root galls compared to the rest of the root [12].

Since the *S. lycopersicum CYP710A11* gene is known to be repressed during *M. incognita* infection, and tomato plants only possess a single copy of it [13], we investigated the temporal regulation of *CYP710A11* during *M. incognita* infection and the changes in the tomato β-sitosterol/stigmasterol composition. Furthermore, we infected tomato mutant lines lacking a functional *CYP710A11* gene and, therefore, lacking stigmasterol with *M. incognita* and evaluated the reproduction of *M. incognita* and its ability to complete its lifecycle in the absence of stigmasterol.

## 2. Results

### 2.1. Characterization of the Micro-Tom cyp710a11 Mutant Line

**Figure 2:**
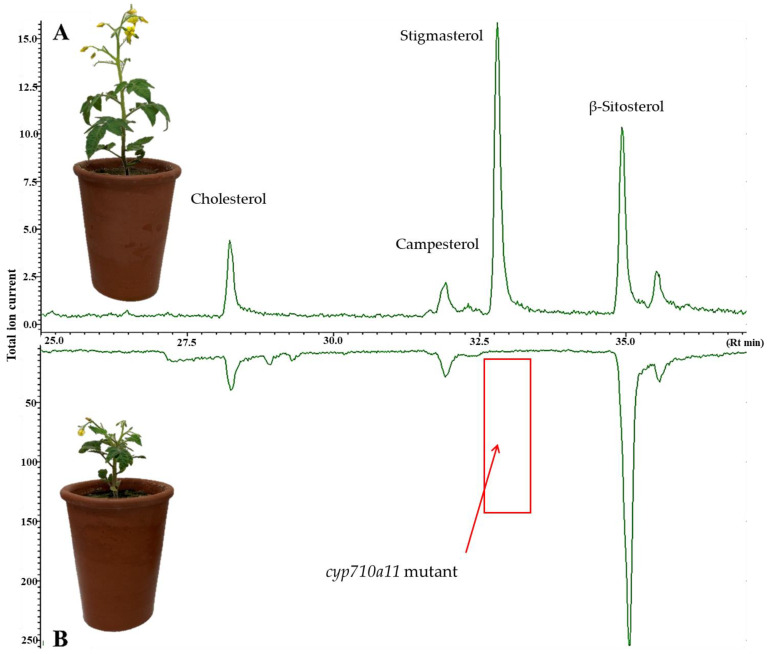
Chemical characterization of the sterol compositions of the wt (**A**) and the *cyp710a11* knockout mutant (**B**) of tomato cv. Micro-Tom by gas chromatography–mass spectrometry. The loss of stigmasterol in the *cyp710a11* knockout mutant plant is indicated by a red box in the representative MS chromatogram shown here (green line).

To evaluate the role of stigmasterol in the plant host’s suitability for *M. incognita*, a sterol C-22 desaturase mutant tomato line of the cultivar Micro-Tom with a T-DNA insertion in the *CYP710A11* gene was obtained from the Tsukuba Plant Innovation Research Center. The developmental trajectories of the wild type (wt) and mutant were compared (Table 1). All the mutant plants showed reduced growth compared to the wt plants (Table 1, Figure 2A,B). The average shoot and root fresh weights were 4.32 g/plant and 3.50 g/plant for the wt, respectively, significantly higher compared to the *cyp710a11* mutant plants, which had an average shoot fresh weight of 2.82 g/plant and a root fresh weight of 2.22 g/plant. In addition, the average wt shoot length was 18.62 cm, significantly longer than the shoot length of the mutant plants, which was 10.58 cm on average. The shoot dry weight represented 11.57% of the total dry weight for the wt plants compared to 10.92% for the *cyp710a11* mutant plants, showing no significant differences.

To confirm the genotype of the *cyp710a11* mutant plants, the sterol compositions of plants were analyzed after each experiment via gas chromatography–mass spectrometry (GC-MS). The absence of stigmasterol was confirmed for each mutant plant (Figure 2).

### 2.2. Sterol Composition of the Micro-Tom wt and cyp710a11 Mutant Plants after M. incognita Infection

To determine whether the wt tomato cv. Micro-Tom showed changes in its sterol composition similar to those previously reported by Cabianca et al. [12] for the tomato cv. Moneymaker after *M. incognita* infection, a temporal analysis of the composition of the free sterol pool of the roots after *M. incognita* infection was performed for both cultivars.

**Figure 3:**
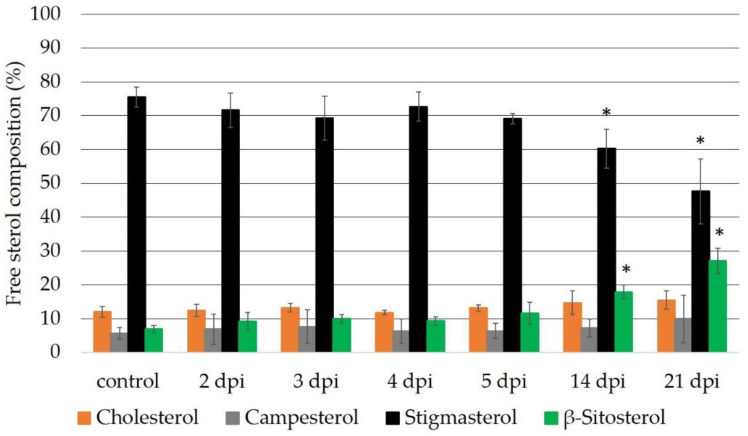
Gas chromatography–mass spectrometry analysis of the composition of the free sterols in the uninfected tomato cv. Moneymaker roots (control) and at 2, 3, 4, 5, 14 and 21 days post-inoculation with *M. incognita* (dpi), respectively. n = 4 biological replicates of 2 pooled plants per analysis. ANOVA was used for comparisons of the time and sterol alterations. *, significantly different from the control at *p* < 0.05.

The sterol composition of the tomato cv. Moneymaker at 2, 3, 4, 5, 14 and 21 days post-inoculation with *M. incognita* (dpi) revealed that the β-sitosterol levels increased over time, while the stigmasterol levels decreased (Figure 3). Significant differences in the levels of β-sitosterol and stigmasterol compared to the control plants were observed after 14 and at 21 days post-inoculation.

**Figure 4:**
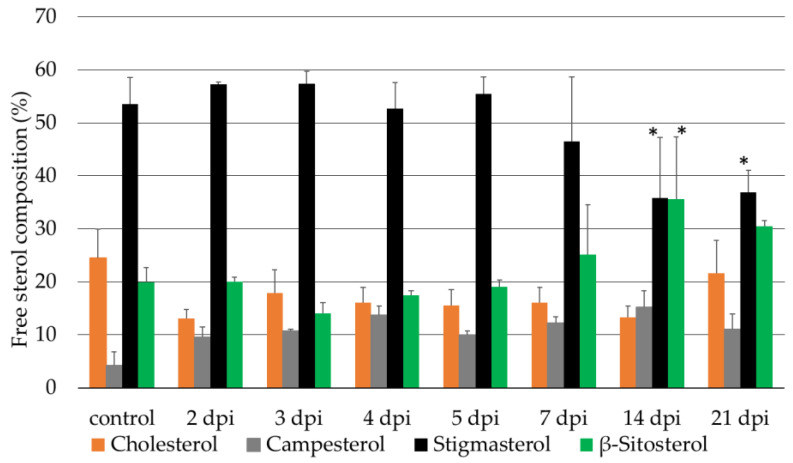
Chemical characterization of the wt tomato cv. Micro-Tom free sterol composition for the uninfected (control) plants and 2, 3, 4, 5, 7, 14 and 21 days post-inoculation with *M. incognita* (dpi) using gas chromatography–mass spectrometry. n = 4 biological replicates of 2 pooled plants per analysis. ANOVA was used for comparisons of the time and sterol alterations. *, significantly different from the control at *p* < 0.05.

The free sterol composition of the wt cv. Micro-Tom was analyzed at the time points 2, 3, 4, 5, 7, 14 and 21 dpi with *M. incognita* (Figure 4). The *M. incognita*-infected tomato cv. Micro-Tom showed sterol changes similar to those of the tomato cv. Moneymaker. A reduction in stigmasterol and increase in β-sitosterol were observed, as in the tomato cv. Moneymaker (Figure 3 and Figure 4). Significant changes in the β-sitosterol to stigmasterol ratio were observed at 14 and 21 dpi, as in the case of cv. Moneymaker. Comparing the free sterol composition in the root systems of both cultivars, cv. Micro-Tom showed higher amounts of β-sitosterol compared to cv. Moneymaker (Figure 3 and Figure 4). The stigmasterol to β-sitosterol ratio was similar to that of cv. Moneymaker in the cv. Micro-Tom plants at 14 dpi and slightly different at 21 dpi. In addition, the cv. Micro-Tom plants had higher cholesterol levels than cv. Moneymaker, and while the cholesterol and campesterol levels were relatively steady following the inoculation with *M. incognita* for cv. Moneymaker, they oscillated under the same conditions in cv. Micro-Tom.

Furthermore, the free sterol analysis of the *cyp710a11* mutant plants infected with *M. incognita* showed that, due to the absence of stigmasterol, the contribution of β-sitosterol to the free sterol pool in the roots and particularly in the leaves was significantly higher than in the wt (Table 2). As expected, based on the biosynthetic pathway, the lack of stigmasterol did not affect the contributions of campesterol and cholesterol to the sterol pool in either the roots or leaves. It should be noted that, in both lines, the cholesterol levels were lower in the leaves compared to the roots, while the campesterol levels were similar in the roots and leaves.

### 2.3. Effect of M. incognita on Micro-Tom cyp710a11 Mutant Plants

The tomato root gall index (GI), *M. incognita* egg mass count and egg count were evaluated for the Micro-Tom wt and *cyp710a11* mutant plants so as to evaluate whether the loss of CYP710A11 affected the defense against, or the ability to host, *M. incognita*. In two consecutive experiments, no statistical difference was observed between the wt Micro-Tom and *cyp710a11* mutant plants regarding the GI (Table 3). The average GI was 6.67 ± 0.48 and 6.43 ± 0.51 for the wt plants and 6.13 ± 0.67 and 7.00 ± 0.60 for the mutant line lacking stigmasterol in the first and second experiments, respectively.

**Figure 5:**
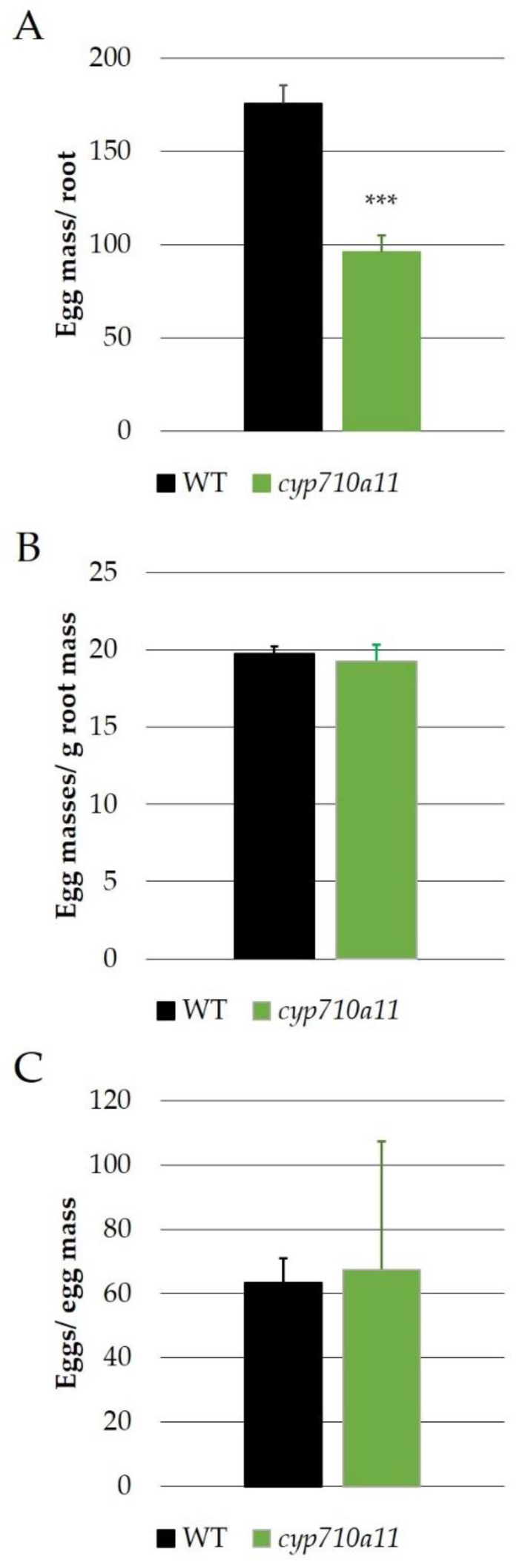
*M. incognita*’s effect on the cv. Micro-Tom wt and *cyp710a11* knockout mutant tomato plants is shown as the egg mass per root (**A**), egg mass per g of root mass (**B**) and number of eggs per egg mass (**C**). n = 6 biological replicates. Statistical significance was calculated using a two-tailed Student’s *t*-test (*** significantly different from wt at *p* ≤ 0.001).

The *M. incognita* egg masses were counted in the wt and mutant roots, showing a significantly higher amount of egg masses in the wt plants (175.6 egg masses/root) compared to the mutant line (95 egg masses/root) (Figure 5A). However, when the egg masses per g root weight were quantified, there was no significant difference between the wt and mutant line (19.76 egg masses/g of root for wt and 19.29 egg masses/g of root for the mutant; Figure 5B). Similarly, no significant differences were observed for the egg to egg mass ratio, with 63.69 eggs/egg mass for Micro-Tom and 67.49 eggs/egg mass for the *cyp710a11* mutant plants (Figure 5C).

**Figure 6:**
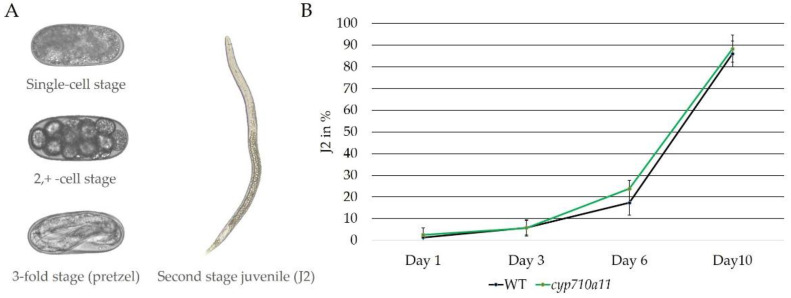
*M. incognita* development from an egg to second-stage juvenile (J2), extracted from the wt cv. Micro-Tom or *cyp710a11* mutant tomato plants. The egg development was recorded over 10 days (Figure S2). The single-cell stage, 2, + − cell stage, 3-fold stage (pretzel stage) and second stage juvenile (J2) were counted (**A**). The development of the J2 stage on days 1, 3, 6 and 10 is presented as the percentage of J2 in the total egg count (**B**). n = 6 biological replicates. No significant differences were recorded using a two-tailed Student’s *t*-test.

*M. incognita*’s development from an egg to second-stage juvenile (J2) was further examined for the eggs extracted from the wt and *cyp710a11* mutant tomato cv. Micro-Tom plants. The egg’s development from the single-cell stage through the 2, + − cell stage to the 3-fold stage and then to a J2 showed no differences over a 10-day period whether eggs were extracted from the wt or from the *cyp710a11* mutant plants (Figure 6 and Figure S1).

The root growth of the wt and the mutant line infected with *M. incognita* was compared using the open-source software RhizoVision Explorer v2.0.3 [19] (Figure S3). The root growth of the knockout mutant *cyp710a11* was reduced to approximately 67% of that of the wt roots. For example, the total root length per wt root system of 95,996.17 mm was significantly longer than the length of 64,667.00 mm per root system in the *cyp710a11* mutant plants. Relative to the root length, the *cyp710a11* mutant had a lower infection rate than the wt plants, with one egg mass per 670.41 mm of the mutant root length versus one egg mass per 545.62 mm of the wt root length, respectively (Table 4). Other parameters were also recorded and are presented in Table S1. However, no further significant differences were found between the wt and mutant line.

## 3. Discussion

The Micro-Tom *cyp710a11* mutant line showed, in addition to a lack of stigmasterol, reduced root and shoot growth, which is noteworthy, as studies on the corresponding stigmasterol-free maize mutant, *cyp710a8*, showed no growth phenotype [20]. Transgenic Arabidopsis lines containing high amounts of stigmasterol and low amounts of sitosterol due to overexpression of *CYP710A8* genes from wheat or barley did not show any growth phenotype either [21]. However, Arabidopsis plants supplemented exogenously with either sitosterol or stigmasterol showed an increased expression of genes associated with cell expansion and cell division [22], which might indicate a link between their growth and sterol production. The exogenous application of stigmasterol improved the growth of basil plants [23] and wheat, presumably via the activation of the anti-oxidant defense system [24]. However, this does not explain the observed growth phenotype of Micro-Tom *cyp710a11*. Interestingly, studies on an Arabidopsis sterol methyltransferase mutant, *smt1*, showing decreased sitosterol and stigmasterol levels combined with increased cholesterol levels, resulted in the disturbed localization of the PIN proteins, auxin efflux transporters [25]. However, while exogenously supplied auxin does increase plant growth, in general, and the growth of Micro-Tom, in particular [26], the broad effects on PIN localization can be expected to disturb the plant’s development, as it does in the Arabidopsis *sm1* mutant. Altogether, the growth phenotype of Micro-Tom *cyp710a11* cannot be explained based on the literature and might be due to a second T-DNA insertion.

Sterol C-22 desaturase encoded by *CYP710A11* has been reported to play a role in biotic and abiotic stress in plants by altering the ratio of β-sitosterol to stigmasterol [20]. Furthermore, infection by *M. incognita* led to a reduction in the contribution of stigmasterol to the sterol pool in tomato, as well as cotton, and to the downregulation of the expression of the tomato gene *CYP710A11* (Figure S1) [11,12]. Consequently, further studies have been conducted to help us to understand the role of the tomato sterol C-22 desaturase in *M. incognita* infection.

The temporal expression analysis of the *CYP710A11* gene following the *M. incognita* infection of cv. Moneymaker confirmed its eventual suppression at 3 dpi (Figure S1). Our results validated the observation that the tomato cultivar Micro-Tom showed the same behavior as cvs. Oskar and Moneymaker [12]. Thus, infection with *M. incognita* suppressed stigmasterol production in the tomato cultivars Oskar, Moneymaker and Micro-Tom, and a strong correlation could be observed between the decrease in the *CYP710A11* mRNA levels and the decrease in the stigmasterol levels ca. eleven days later (Figure 3, Figure 4 and Figure S1). Therefore, we suggested that the reduction in stigmasterol production is controlled at the *CYP710A11* transcription level, as in tomato, this sterol C-22 desaturase is encoded by a single gene, in contrast to Arabidopsis [13,27], rice [28] or poplar [27], which contain multiple copies. We further assumed that the changes in the C-22 desaturase levels represented an active response to *M. incognita* infection. However, whether this response is induced specifically by the nematode or represents a plant response to biotic stress in general remains to be seen. The latter is unlikely, as the opposite effects have been reported for infections caused by other pathogens. For example, infection with *Pseudomonas syringae* or *Golovinomyces cichoracearum* increased the expression of *CYP710A* in *Arabidopsis thaliana* [29,30,31]. In cotton (*Gossypium hirsutum*), infection with the fungal pathogen *Verticillium dahliae* led to the upregulation of the expression of *GhCYP710A1*, increasing the ratio of stigmasterol to β-sitosterol [32]. In contrast, *M. incognita* infection in cotton led to a decrease in the ratio of stigmasterol to β-sitosterol [11]. Altogether, these data suggest that the induction of sterol C-22 desaturase expression is not a common plant response to pathogens, but that different plants respond in distinct ways to different pests and pathogens, and the reduction in stigmasterol biosynthesis is a specific plant response to *M. incognita*.

The analysis of the tomato cv. Micro-Tom wt and of the *cyp710a11* mutant plants lacking stigmasterol, aiming to assess the function of the tomato *CYP710A11* gene and its role in *M. incognita*-plant interactions, showed that the mutant plants, despite their reduced growth, showed a similar root gall rating to the wt plants. Although the smaller *cyp710a11* mutant plants showed a reduced number of *M. incognita* egg masses per plant, there were no significant differences in the egg mass counts relative to the root weight compared to the wt. Still, the computing algorithms used to analyze the images of the root systems (the software RhizoVision Explorer v2.0.3 [19]) showed that the wt plants had more egg masses per root length (mm) compared to the mutant plants.

Due to the reduced growth of the mutant plants, *M. incognita* propagation was impaired. However, the development of the *M. incognita* eggs grown on the wt and *cyp710a11* loss-of-function mutant plants showed no differences. Within 10 days, most eggs from wt or mutant plants reached the J2 stage (Figure 6). Overall, the development of *M. incognita* in the *cyp710a11* mutant plants suggests that the nematode did not require plant stigmasterol.

The fact that *M. incognita* can cope with the lack of stigmasterol may be due to different reasons. *M. incognita* has the ability to propagate on plants with different compositions of the root sterols, an ability that might be required for a polyphagous nematode. For instance, crops such as *Brassica juncea* naturally have very low stigmasterol levels [12]. Thus, *M. incognita* may not depend on specific C-24-phytosterols for its development. Cholesterol, in particular, is reported to be essential for nematodes [33,34]. Recently, the study of a reconfigured sterol modification pathway in *C. elegans* suggested that stigmasterol (plant) or ergosterol (fungi) can be converted into cholesterol [7]. However, this sterol modification pathway remains to be elucidated in the case of plant parasitic nematodes. Given that *C. elegans* requires only small amounts of cholesterol in its diet [35], and the free sterol pool of the cv. Micro-Tom wt and the *cyp710a11* mutant contained more than 10% cholesterol, we hypothesize that *M. incognita* developed using cholesterol.

Interestingly, the contribution of cholesterol to the free sterol pool of the infected plants did not differ greatly from that of the uninfected plants. These findings are of interest, as Solanaceous plants, such as potato (*Solanum tuberosum*) and tomato, synthesize steroid glycoalkaloids from cholesterol in response to abiotic and biotic stress [36,37,38,39,40].

Due to the importance of sterols for the nematodes, additional investigations of sterols such as cholesterol, campesterol and β-sitosterol should be performed as a potential approach to controlling plant parasitic nematodes. After all, similar approaches have been successful in the case of herbivorous insects, which also lack the ability to synthesize their own sterols [6]. Plant sterol constraints have been exploited as a novel strategy to control insect herbivores by altering the sterol composition of tobacco plants to include high levels of atypical plant steroids (stanols and 3-ketosteroids). Three economically important caterpillars, *Heliothis virescens*, *Spodoptera exigua* and *Manduca sexta* [41], as well as the phloem-feeding green peach aphids (*Myzus persicae*), have shown reduced survival, growth and fecundity on these plants [10].

## 4. Materials and Methods

### 4.1. Nematode Inoculum

The root-knot nematodes *M. incognita* were maintained on tomato *Solanum lycopersicum* cv. Oskar grown under greenhouse conditions at 22 ± 2 °C, with a light/dark 16 h/8 h rhythm and 60% relative humidity, at Agroscope (Wädenswil, Switzerland). Heavily galled root systems were placed in a mist chamber to extract the second-stage juveniles (J2) [42]. The extracted J2 were stored at 6 °C for no longer than 10 days before use.

### 4.2. Tomato cv. Micro-Tom cyp710a11 Mutant Line

Seeds of the T-DNA-tagging Micro-Tom line (TOMJPT00087-2) with the T-DNA insertion site of the *CYP710A11* gene (Solyc02g070580; hit region SL3.0ch02:40877249. .40877014), were provided by the Tsukuba Plant Innovation Research Center, University of Tsukuba, through the National Bio-Resource Project (NBRP) of the Japan Agency for Research and Development (AMED), Tsukuba, Japan.

The Micro-Tom wt and *cyp710a11* mutant line were germinated for two weeks in potting soil. The germinated seedlings were subsequently transplanted into 14 cm-diameter pots filled with a 3:1 (*v*/*v*) silver sand:steamed soil mixture and grown at 24 °C with a light/dark rhythm of 16 h/8 h and 60% relative humidity. The roots and shoots of the eight-week-old wt and mutant plants were separated. The roots were washed free of soil and dried to remove the excess water in order to weigh the root fresh weight in g. The shoots were measured (length in cm), and their weight (including leaves) was measured to determine the shoot fresh weight in g. The cut shoots were placed in a drying oven at 70 °C, and their weight was determined daily until no weight change was recorded. Subsequently, the root and shoot dry weight was determined as a percentage of the total dry weight.

### 4.3. CYP710A11 Gene Expression Analysis

Temporal gene expression analyses of the tomato gene *CYP710A11* were conducted by challenging the three-week old tomato plants of cv. Moneymaker with 6000 *M. incognita* J2/plant. Tomato roots were harvested from the uninfected (control) and infected plants at 2, 3, 4, 5, 14 and 21 days post-inoculation with *M. incognita* (dpi), frozen in liquid nitrogen and ground into powder. An aliquot (100 mg) of the powder was used for the RNA extraction using the Thermo Scientific GeneJET Plant RNA Purification Mini Kit (Waltham, MA, USA). The RNA was quantified, and its purity was assessed using a NanoDrop One/OneC Microvolume UV–Vis Spectrophotometer (Thermo Fisher Scientific, Reinach, Switzerland). cDNA synthesis was performed using the iScript cDNA synthesis kit (Bio-Rad, Hercules, CA, USA). The tomato *CYP710A11* gene primers from Cabianca et al. [12] were used for the qPCR analysis. GoTaq qPCR Master Mix from Promega (Madison, WI, USA) was used to perform the qPCR analysis using a Roche LightCycler 480 (Rotkreuz, Switzerland). The Roche LightCycler 480 program was used for the melting curve and temperature analysis.

### 4.4. Sterol Extraction and Gas Chromatography–Mass Spectrometry Analysis

Aliquots (2 g) from the Micro-Tom plants or Moneymaker plant tissue powder used for the gene expression analysis were used for the sterol extraction. The total lipids were extracted using chloroform:methanol (2:1, *v*/*v*) for 1 h at 60 °C, according to Blight and Dyer [43]. The lipid fraction separated from each root sample was dried under nitrogen gas. The dried samples were resuspended in hexane, and the sterols were separated using a silica solid phase extraction (SPE) column (6 mL SiOH columns, Chromabond, Macherey Nagel, Düren, Germany), according to Azadmard-Damirchi and Dutta [44]. The sterol fractions were dried under nitrogen and resuspended in the chloroform that was used as solvent for GC-MS analysis using a Varian 450-GC coupled with a Varian 240-MS Ion Trap (Darmstadt, Germany). The VARIAN FactorFour capillary column VF-5 ms with a 30 m length, 0.25 mm inner diameter and 0.25 µm film thickness was used as the stationary phase, and the GC-MS settings were applied using the VARIAN MS Workstation v.6.9.3 (Walnut Creek, CA, USA), as described by Cabianca et al. [12]. The cholesterol, campesterol, β-sitosterol and stigmasterol standards were obtained from Sigma-Aldrich (St. Louis, MO, USA).

### 4.5. M. incognita Growth on the Tomato cv. Micro-Tom cyp710a11 Mutant Line

The Micro-Tom wt and sterol C-22 desaturase mutant lines were germinated for two weeks in potting soil before being transplanted into 14 cm-diameter pots filled with a 3:1 (*v*/*v*) silver sand:steamed soil mixture and grown at 24 °C with a light/dark rhythm of 16 h/8 h and 60% relative humidity. Then, the 4-week-old mutant and wt plants were challenged with 6000 *M. incognita* J2 per plant (n = 6). Four weeks after nematode inoculation, the roots were gently washed to remove the soil, and the root galling was indexed according to Zeck [18], and the egg masses were stained with Ponceau 4R (Thermo Fisher Scientific, India), a red food colorant, as described by Hallmann et al. [45].

Eggs were extracted from the root by cutting the root system into 1 cm pieces and shaking them in 1.5% bleach solution (NaClO, the concentration refers to the active ingredient) for 3 min. The eggs were separated from the root debris by filtering the solution through 200 µm, 100 µm, 75 µm and 50 µm stacked sieves and collecting the eggs in a 20 µm sieve. The eggs were counted under an inverted light microscope (40×).

Another set of unstained roots were used to follow the nematode development. The extracted eggs from this set of roots were used to follow the lifecycle of *M. incognita* from the egg to the J2 stage on 6-well plates at 22 °C in the dark. The egg development from the single-cell stage, 2, + − cell stage and 3-fold stage (pretzel stage) to the J2 stage was recorded after 1, 3, 6 and 10 days under an inverted light microscope (40×) by counting 100 eggs or J2 per plant for the wt and *cyp710a11* mutant (n = 6). For each plant, sterols were extracted from the mutant line and wt Micro-Tom and analyzed as described previously.

### 4.6. Comparison of the Root Development of the Tomato cv. Micro-Tom wt and cyp710a11 Mutant Lines Infected with M. incognita

Root systems images were taken against a dark background and converted to all-negative channels using the freeware IrfanView v4.56 (Wiener Neustadt, Austria). Subsequently, the open-source software RhizoVision Explorer v2.0.3 [19] was used to analyze the root images using the algorithms described by Seethepalli et al. [46].

### 4.7. Statistical Analysis

The software R (v. 3.6.2; R core team, 2018; Vienna, Austria) was used to perform Student’s *t*-tests (*t*-tests) and an analysis of variance (ANOVA) using the data obtained to investigate the statistical differences between the samples. Samples with a significance level of *p* ≤ 0.05 were considered statistically different. Graphs were designed using R or MS Excel.

## 5. Conclusions

Based on our findings, we can confirm that the *CYP710A11* gene expression levels are downregulated in different tomato cultivars after *M. incognita* infection, leading to a reduction in the stigmasterol levels and an increase in the β-sitosterol levels in the roots. The downregulation of the *CYP710A11* gene is a direct response of the plant to the nematode. Based on the comparison with the other interactions, we assume that it is a specific response to the nematode and not a general response to biotic stress.

Stigmasterol does not seem to be crucial for the development of sterol-auxotrophic *M. incognita*, since the nematodes developed successfully in the *cyp710a11* mutant tomato plants in the absence of stigmasterol. While the reduced plant growth led to a reduction in the nematode egg mass count per plant in the mutant compared to the wt, the egg mass count per root weight was not affected. Nevertheless, further research is needed to better understand the physiological role of stigmasterol in plant-nematode interactions. Furthermore, the roles of other sterols (cholesterol, campesterol and β-sitosterol) should be investigated as a potential approach to controlling plant parasitic nematodes.

## Figures and Tables

**Table 1:** Phenotypical data of the wt and the *cyp710a11* knockout mutant of the tomato cv. Micro-Tom.

Micro-Tom	Root Fresh Weight (g)	Shoot Fresh Weight (g)	Shoot Dry Weight in %	Shoot Length (cm)
Wt	3.50 ± 0.50 ^a^	4.32 ± 0.53 ^a^	11.57 ^a^	18.62 ± 1.07 ^a^
*cyp710a11*	2.22 ± 0.24 ^b^	2.82 ± 0.39 ^b^	10.92 ^a^	10.58 ± 1.08 ^b^

Samples were taken from eight-week-old plants. The number of replicates was n = 6 for the wt plants and n = 5 for the mutant plants. Statistical significance was calculated using a two-tailed Student’s *t*-test. Means followed by the same letter within a column are not significantly different (*p* < 0.05).

**Table 2:** Gas chromatography–mass spectrometry analysis of the free sterol compositions of the roots and leaves from the tomato cv. Micro-Tom wt and *cyp710a11* mutant infected with *M. incognita*. Samples were taken at 28 dpi.

Sterols	Micro-Tom Roots		Micro-Tom Leaves	
	wt	*cyp710a11*	wt	*cyp710a11*
Cholesterol	14.78 ± 1.38 ^a^	14.69 ± 0.74 ^a^	8.99 ± 1.13 ^a^	9.69 ± 0.44 ^a^
Campesterol	13.85 ± 0.46 ^a^	13.09 ± 1.77 ^a^	14.18 ± 1.99 ^a^	16.91 ± 3.13 ^a^
Stigmasterol	43.36 ± 1.37 ^a^	0 ^b^	69.75 ± 2.87 ^a^	0 ^b^
β-Sitosterol	28.01 ± 2.12 ^a^	72.22 ± 2.04 ^b^	7.20 ± 2.76 ^a^	73.39 ± 3.52 ^b^

n = 6 biological replicates for the roots and n = 4 biological replicates for the leaves. Statistical significance was calculated using a two-tailed Student’s *t*-test. Means followed by the same letter within a column are not significantly different (*p* < 0.05).

**Table 3:** Root gall index recorded after *M. incognita* infection of the cv. Micro-Tom wt and the *cyp710a11* knockout mutant plants in two consecutive experiments.

Micro-Tom	GI Experiment 1	GI Experiment 2
Wt	6.67 ± 0.48 ^a^	6.43 ± 0.51 ^a^
*cyp710a11*	6.13 ± 0.67 ^a^	7.00 ± 0.60 ^a^

Root galling (GI) was indexed according to Zeck [18], with 0 = no root galls and 10 = severe galled-up roots. n = 6 biological replicates. Statistical significance was calculated using a two-tailed Student’s *t*-test. Means followed by the same letter within a column are not significantly different (*p* > 0.05).

**Table 4:** Root measurements of the wt and *cyp710a11* mutant of cv. Micro-Tom tomato plants infected with *M. incognita* using RhizoVision Explorer v2.0.3 [19].

Micro-Tom	Median Number of Roots	Max. Number of Roots	Number of Root Tips	Total Root Length (mm)	Egg mass/mm Root
wt	10.80 ± 2.86 ^a^	34.40 ± 6.35 ^a^	2967.20 ± 756.11 ^a^	95,996.17 ± 25,993.51 ^a^	545.62 ± 117.76 ^a^
*cyp710a11*	10.00 ±2.17 ^a^	28.00 ± 3.81 ^a^	2124.83 ± 324.30 ^b^	64,667.00 ± 12,144.3 ^b^	670.41 ± 83.39 ^a^
*p*-value	0.61	0.07	0.03	0.03	0.08

n = 5 or n = 6 biological replicates for the wt and *cyp710a11* mutant cv. Micro-Tom tomato plants, respectively. Statistical significance of the differences was calculated using a two-tailed Student’s *t*-test. Means followed by the same letter within a column are not significantly different (*p* > 0.05).

## Data Availability

Not applicable.

## Supplementary Materials

The following supporting information can be downloaded at: https://www.mdpi.com/article/10.3390/ijms232315111/s1.
