# Supplementary material for: Tomato Sterol 22-desaturase Gene CYP710A11: Its Roles in Meloidogyne incognita Infection and Plant Stigmasterol Alteration
Source: Int J Mol Sci. 2022 Dec 1;23(23):15111. doi: 10.3390/ijms232315111 (PMC9735470; doi:10.3390/ijms232315111)
Supplement: Supplementary file 1 [file ijms-23-15111-s001.zip › ijms-2054507-supplementary.pdf]

Table S1. Root measurements of wt and *cyp710A11* mutant cv. Micro-Tom tomato plants infected with *Meloidogyne incognita*.

Root features	Tomato plants cv. Micro-Tom		Statistical significance
	wt	*cyp710a11*	p-value
Depth (mm)	2523.00 ±156.47 [a]	2078.00 ± 327.87 [b]	0.0219
Maximum width (mm)	1704.40 ± 205.32 [a]	1125.00 ± 256.02 [b]	0.0028
Width-to-depth ratio (mm)	0.68 ± 0.07 [a]	0.55 ± 0.13 [a]	0.0770
Network area (mm^2)	704701.20 ± 174837.71 [a]	436625.50 ± 87196.05 [b]	0.0090
Convex area (mm2)	2962785.60 ± 695214.03 [a]	1701601.25 ± 423038.86 [b]	0.0048
Solidity	0.25 ± 0.06 [a]	0.26 ± 0.04 [a]	0.7482
Lower root area (mm^2)	574975.00 ± 321669.14 [a]	355491.17 ± 15543.23 [a]	0.1712
Average diameter (mm)	14.42 ± 1.56 [a]	12.95 ± 1.65 [a]	0.1660
Median diameter (mm)	10.53 ± 0.67 [a]	9.40 ± 1.03 [a]	0.0650
Maximum diamter (mm)	96.94 ± 13.24 [a]	78.64 ± 18.75 [a]	0.1008
Perimeter (mm)	96125.98 ± 22509.65 [a]	67095.22 ± 12513.50 [b]	0.0239
Volume (mm^3)	28037777.76 ± 9310200.21 [a]	15181545.73 ± 4593999.18 [b]	0.0151
Surface area (mm^2)	4419095.53 ± 1243264.28 [a]	2684299.64 ± 561094.05 [b]	0.0130
Holes	1473.60 ± 620.63 [a]	1106.50 ± 420.69 [a]	0.2729
Average hole size (mm^2)	556.32 ± 234.24 [a]	491.27 ± 289.88 [a]	0.6964
Average root orientation (deg)	45.01 ± 0.86 [a]	45.54 ± 1.34 [a]	0.4667
Shallow angle frequency	0.32 ± 0.02 [a]	0.32 ± 0.02 [a]	0.8724
Medium angle frequency	0.35 ± 0.02 [a]	0.34 ± 0.01 [a]	0.1229
Steep angle frequency	0.33 ± 0.01 [a]	0.35 ± 0.02 [a]	0.1636

RhizoVision Explorer v2.0.3 (Seethepalli and York, 2020) was used to analyze the root images using the algorithms described by Seethepalli et al. [46]. N = 5 or 6 biological replicates for wt and *cyp710a11* mutant cv. Micro-Tom tomato plants, respectively. Statistical significance was calculated using a two-tailed Student t-test. Means followed by the same letter within the same line are not significantly different ($p > 0.05$).

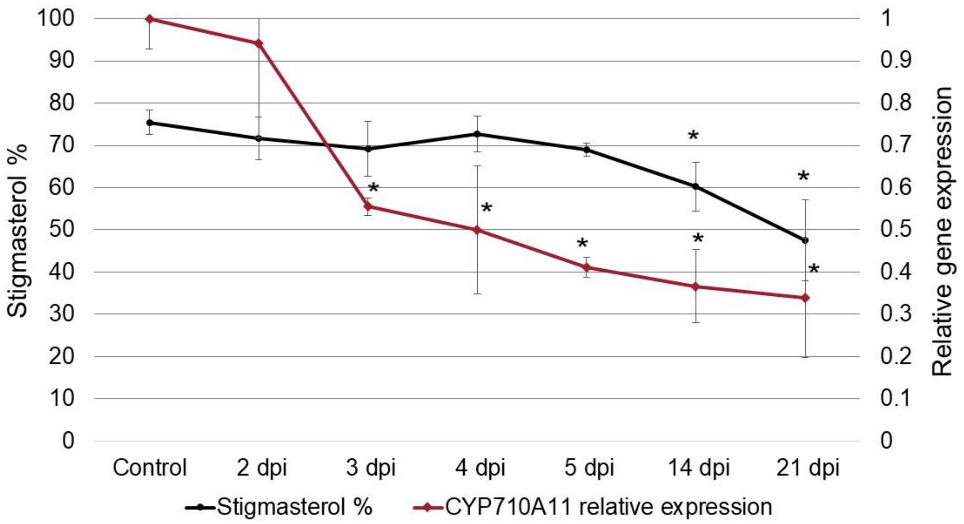

Figure S1. Root temporal gene expression analysis of the tomato cv. Moneymaker C-22 desaturase gene *CYP710A11* and the relative stigmasterol concentration compared to the overall free sterol composition in percentage of the uninfected control plants, at 2, 3, 4, 5, 14 and 21 days post inoculation with *Meloidogyne incognita* (dpi). N = 4 biological replicates of 2 pooled plants each per analysis. ANOVA was used for comparison of time and sterol content alterations. *, significantly different from control with $p < 0.05$.

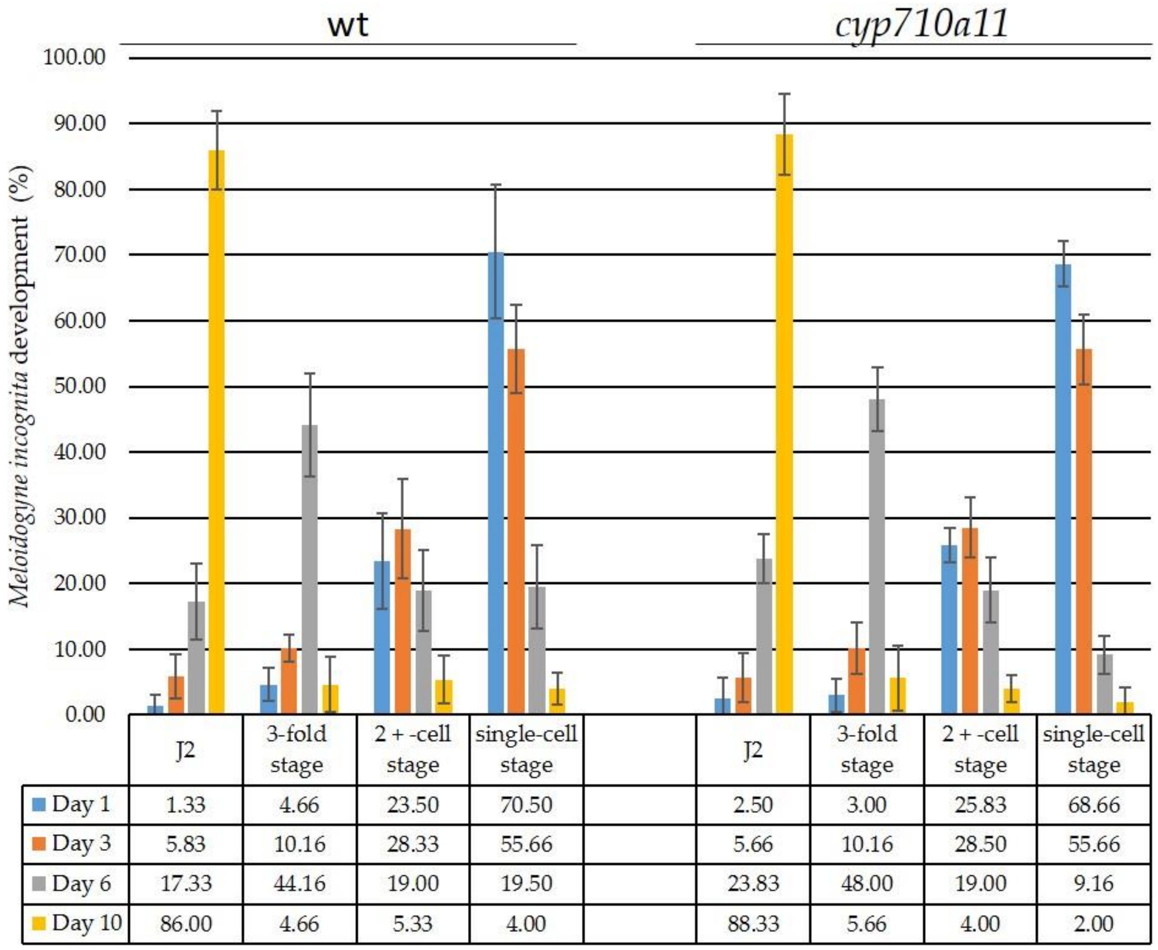

	wt: J2	wt: 3-fold stage	wt: 2 + -cell stage	wt: single-cell stage	*cyp710a11*: J2	*cyp710a11*: 3-fold stage	*cyp710a11*: 2 + -cell stage	*cyp710a11*: single-cell stage
Day 1	1.33	4.66	23.50	70.50	2.50	3.00	25.83	68.66
Day 3	5.83	10.16	28.33	55.66	5.66	10.16	28.50	55.66
Day 6	17.33	44.16	19.00	19.50	23.83	48.00	19.00	9.16
Day 10	86.00	4.66	5.33	4.00	88.33	5.66	4.00	2.00

Figure S2. *Meloidogyne incognita* development from single-cell stage, 2 + -cell stage, 3-fold stage (pretzel stage) to second stage juvenile (J2), extracted from wt or *cyp710a11* mutant tomato plants cv. Micro-Tom, for days 1, 3, 6 and 10 presented as percentage. n = 6 biological replicates.

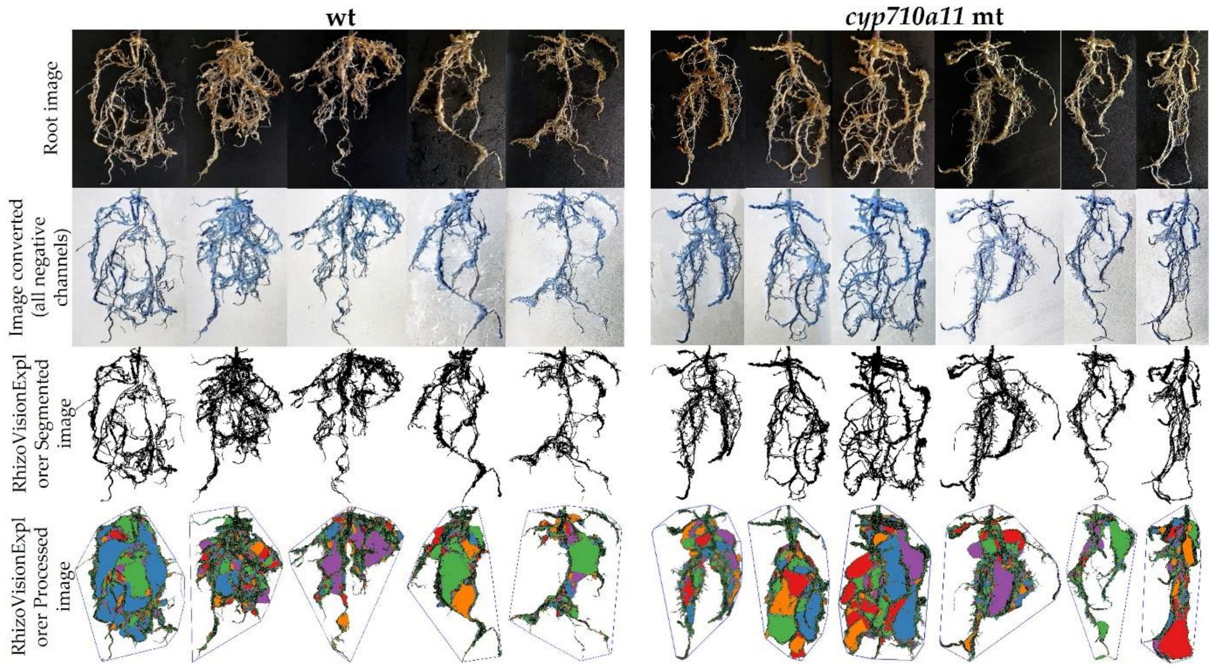

Figure S3. Root images from wt and *cyp710a11* mutant of cv. Micro-Tom tomato plants infected with *Meloidogyne incognita* used for root measurements (Tables 4 and S1) and its processed images created by RhizoVision Explorer v2.0.3 [19].
